# MDCO-216 Does Not Induce Adverse Immunostimulation, in Contrast to Its Predecessor ETC-216

**DOI:** 10.1007/s10557-017-6746-x

**Published:** 2017-08-26

**Authors:** Joannes A. A. Reijers, D. G. Kallend, K. E. Malone, J. W. Jukema, P. L. J. Wijngaard, J. Burggraaf, M. Moerland

**Affiliations:** 1 Centre for Human Drug Research, Zernikedreef 8, 2333CL Leiden, The Netherlands; 2 Department of Rheumatology, Leiden University Medical Center, Leiden, The Netherlands; 3 The Medicines Company, Zürich, Switzerland; 4 Good Biomarker Sciences, Leiden, The Netherlands; 5 Present Address: Janssen Prevention Center, Janssen Vaccines and Prevention B.V, Leiden, The Netherlands; 6 Department of Cardiology, Leiden University Medical Center, Leiden, The Netherlands; 7 The Medicines Company, Parsippany, NJ USA

**Keywords:** Immunostimulation, MDCO-216, ETC-216, Ex vivo stimulation, Apolipoprotein A-I Milano

## Abstract

**Purpose:**

Aim of this study was to demonstrate that MDCO-216 (human recombinant Apolipoprotein A-I Milano) does not induce adverse immunostimulation, in contrast to its predecessor, ETC-216, which was thought to contain host cell proteins (HCPs) that elicited an inflammatory reaction.

**Methods:**

Data were taken from a clinical trial in which 24 healthy volunteers (HV) and 24 patients with proven stable coronary artery disease (sCAD) received a single intravenous dose of MDCO-216, ranging 5–40 mg/kg. Additionally, whole blood from 35 HV, 35 sCAD patients and 35 patients requiring acute coronary intervention (aCAD group) was stimulated ex vivo with MDCO-216 and ETC-216.

**Results:**

No inflammatory reaction was observed in HV and sCAD patients following MDCO-216 treatment, judging by body temperature, white cell counts, neutrophil counts, C-reactive protein, circulating cytokines (IL-6, TNF-α), and adverse events. In the ex vivo experiment, the geometric means (SD) of the ratio of MDCO-216 stimulated IL-6 over background levels were 0.8 (1.9), 0.7 (1.5), 1.0 (2.0) for respectively HV, sCAD, aCAD. The corresponding ETC-216 stimulated values were 15.8 (2.9), 9.5 (3.6), 3.8 (4.0). TNF-α results were comparable. Because many ETC-216 stimulated samples had cytokine concentrations >ULOQ, ratios were categorised and marginal homogeneity of the contingency table (MDCO-216 versus ETC-216) was assessed with the Stuart-Maxwell test. *P*-values were ≤0.0005 for all populations.

**Conclusions:**

MDCO-216 did not induce adverse immunostimulation in HV and sCAD patients, in contrast to ETC-216. Results from the ex vivo stimulation suggests the same holds true for aCAD patients.

## Introduction

Over the past decades high density lipoprotein (HDL) and Apolipoprotein A-I (ApoA-I) have been targeted in the pursuit of therapies that reduce the risk of cardiovascular events [1]. One of these therapies is ApoA-I Milano (ApoA-I_M_), a naturally occurring mutant of ApoA-I which was found to be associated with cardioprotective effects [2, 3].

Because of these effects, a human recombinant ApoA-I_M_, codenamed ETC-216, was developed by Esperion Therapeutics in the nineties. In vitro results with recombinant ApoA-I_M_ demonstrated enhanced reverse cholesterol transport, and in animal models regression of atherosclerotic plaques was observed [4].

ETC-216 induced profound lipid changes in the initial phase I study (unpublished results), resulting in a lipid profile that closely resembled carriers of the ApoA-I_M_ mutation. However, dose-dependent increases in neutrophils, paralleling decreases in lymphocytes were observed as well. This phenomenon was first seen in males at a dose level of 50 mg/kg (neutrophil increase >200%), and in females at a dose level of 15 mg/kg (increase ~80%).

**Fig. 1:**
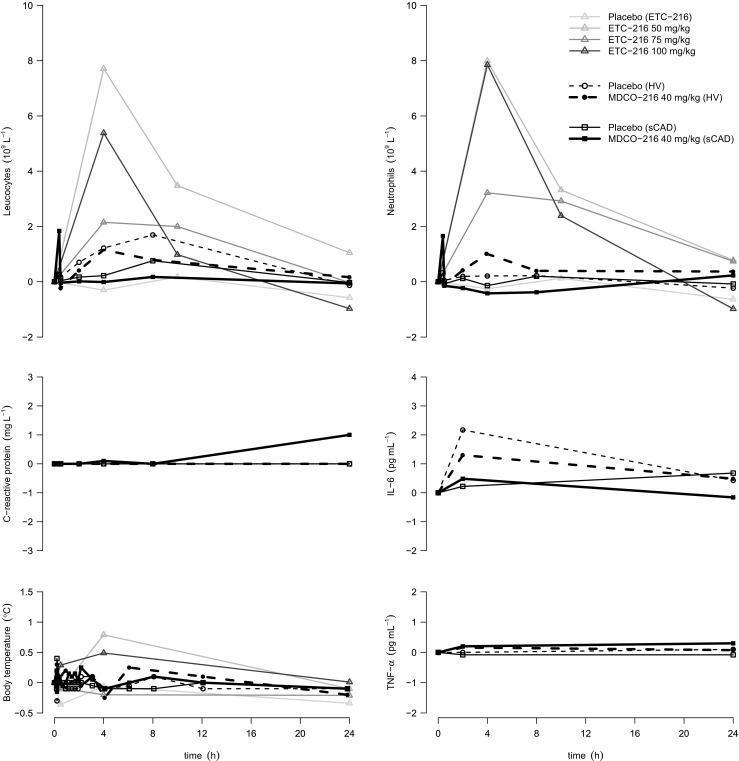
In vivo results. Absolute change (median) over time in clinical markers of inflammation is displayed for placebo and the highest dose (40 mg/kg) of MDCO-216 in healthy volunteers (HV) and in patients with stable coronary artery disease (sCAD). As a reference, the median profiles over time in males in the placebo and highest dose groups from the (unpublished) phase I ETC-216 trial are projected in the background. Of note, 75 mg/kg and 100 mg/kg of ETC-216 were administered at half the infusion rate as was 50 mg/kg of ETC-216 (see main body). Oral temperature served as body temperature in the ETC-216 phase I trial, whereas tympanic membrane temperature was used in the MDCO-216 phase I trial

After decreasing the infusion rate from 1.67 mg/kg/min for males and females to 1.25 mg/kg/min for males and 0.83 mg/kg/min for females, a dose of 100 mg/kg in males and a dose of 50 mg/kg in females was required to induce equal changes in neutrophils and lymphocytes (Fig. 1). Maximum change from baseline in WBC counts was observed at 4 h after the start of the infusion, returning to baseline twenty hours later.

A similar dose-dependent pattern was seen for the adverse events reported within 24 h of administration. Following 15 mg/kg ETC-216 in healthy females, and following 50 mg/kg ETC-216 in healthy males respectively 2/3 and 1/3 participants developed gastrointestinal symptoms (nausea, vomiting, diarrhoea). Lowering the infusion rate reduced the incidence of these events; however, at 100 mg/kg 3/3 participants reported gastrointestinal symptoms. These events occurred approximately 2–4 h after infusion and were sometimes accompanied by systemic symptoms, such as diaphoresis and changes in body temperature.

In the subsequently executed phase II study in patients with an acute coronary syndrome (ACS) [5], it was shown that doses up to 45 mg/kg ETC-216 were associated with a significant regression of the atherosclerotic burden. Not many adverse events were reported, although in a single patient a possible hypersensitivity reaction was noted, consisting of gastrointestinal complaints, rash, chills, and diaphoresis.

Despite the overall promising results, clinical development was halted after a serious adverse reaction had occurred early during the third clinical trial in one patient. When administered intravenously within the hour after percutaneous coronary intervention (PCI), a patient developed a severe reaction during infusion, consisting of flushing, chills, hypotension, ultimately leading to multi-organ failure.

Because the available data pointed to a systemic inflammatory reaction, contamination of the infused drug product was considered. Careful evaluation of the entire manufacturing process revealed that ETC-216 contained small quantities of residual host cell proteins (HCP) that elicited an immune response (unpublished results). Importantly, these effects remained undetected during preclinical development, and became apparent only when the drug was administered to humans, even though all appropriate standards and guidelines had been followed.

A component of the HCP in the ETC-216 drug product was demonstrated to be flagellin, using an assay based on the human toll-like receptor (TLR)-5 [6]. Other impurities included an oligopeptide binding protein (OppA), a dipeptide binding protein (DppA), and maltose-binding periplasmic protein (MalE) [7].

Due to the physical characteristics of ApoA-I_M_ reducing these impurities proved difficult and was ultimately achieved by selectively deleting the genes encoding some of the contaminating proteins and by other significant improvements to the downstream manufacturing process [7–9]. Hereafter, recombinant ApoA-I_M_ was reintroduced as MDCO-216 by the Medicines Company and was tested in healthy volunteers and in patients with stable coronary artery disease (CAD) [10].

This article describes the results of ex vivo stimulation of whole blood samples with ETC-216 and MDCO-216 in three populations (healthy volunteers, patients with stable CAD, and patients with acute CAD). The aim was to characterise the differences in cytokine releasing potential between both drug products. To confirm that the HCPs that induced an inflammatory response were indeed successfully eliminated, the in vivo experience with MDCO-216 was compared to the ex vivo results.

## Methods

### Populations

Table 1 summarises the inclusion criteria of the investigated populations; these encompass both the in vivo and ex vivo exposed populations. Data on the in vivo experience with MDCO-216 came from a randomised, double-blind phase I clinical trial [10], in which 24 healthy volunteers (HV) and 24 patients with stable CAD (sCAD) received a single intravenous dose of 5–40 mg/kg MDCO-216 or placebo.

**Table 1:** Investigated populations

	HV	sCAD	aCAD
Inclusion criteria			
Age (years)	18–55	45–80	≥18
Body weight (kg)	≤110	≤110	–
BMI (kg/m^2^)	18–25	≤40	–
Coronary event	–	requiring a revascularisation procedure	requiring an acute revascularisation procedure
Latency between event and exposure	–	≥1 year	immediately prior to the revascularisation procedure
Concomitant therapy	not allowed, except for oral contraceptives	standard of care, except for HDLc-raising therapy	standard of care
Exposure details			
In vivo	single dose MDCO-216 5–40 mg/kg (*n* = 16) or placebo (*n* = 8) in 2 h	single dose MDCO-216 10–40 mg/kg (*n* = 16) or placebo (*n* = 8) in 2 h	–
Ex vivo	ETC-216 0.5 mg/mL MDCO-216 0.5 mg/mL	ETC-216 0.5 mg/mL MDCO-216 0.5 mg/mL	ETC-216 0.5 mg/mL MDCO-216 0.5 mg/mL

Inclusion criteria for the different populations and details of in and ex vivo exposure. *aCAD* Patients with acute coronary artery disease (CAD); *BMI* Body mass index; *HV* Healthy volunteers; *sCAD* Patients with stable coronary artery disease (CAD)

All subjects enrolled in this trial were also challenged ex vivo with both ETC-216 and MDCO-216, together with those screened for participation and meeting the criteria as listed in Table 1. All trial participants provided written informed consent.

Acute CAD (aCAD) patients were recruited from patients with an acute coronary syndrome (ACS) who presented to the department of cardiology of the Leiden University Medical Center (Leiden, The Netherlands) for a percutaneous coronary intervention (PCI). After verbal approval the blood sample for ex vivo stimulation was collected alongside the routine clinical samples. Written consent was asked at a later stage; if not provided, the blood sample was destroyed and not analysed.

Approval was obtained from independent ethics committees for all trials and related procedures prior to the start of the respective studies, in accordance with pertaining legal requirements.

### Ex Vivo Exposure

Ex vivo stimulations were performed at Good Biomarker Sciences (GBS, Leiden, The Netherlands). Heparinised whole blood samples (18 mL, BD Vacutainer, Becton Dickinson, Breda, The Netherlands) were collected and incubated for 4 h at 37 °C in a humidified atmosphere with 5% CO_2_, before flash-freezing the supernatant for subsequent analysis. Tested conditions were 0.5 mg/mL ETC-216 (The Medicines Company, Zürich, Switzerland), 0.5 mg/mL MDCO-216 (The Medicines Company, Zürich, Switzerland), 2 ng/mL lipopolysaccharide (LPS) gel extracted from *E. coli* serotype O111:B4 (Sigma-Aldrich, St. Louis MO, USA), blanc (unstimulated control). The concentration of 0.5 mg/mL for ETC-216 and MDCO-216 correlates with the maximum plasma concentration achieved following an in vivo dose of 20–30 mg/kg. LPS concentration is based on the EC_90_ of maximum TNF-α release and serves as a positive control. Conditions were made in RPMI with 25 mM HEPES and L-glutamine (GIBCO products from Life Technologies Europe, Bleiswijk, the Netherlands). Final dilution of whole blood required to achieve the tested concentrations was 10%.

Ex vivo exposure of a subject to MDCO-216 always occurred prior to any in vivo exposure (HV and sCAD). Blood samples were kept at 37 °C and processed within one hour after collection, with the exception of the aCAD population, where an interval of up to 12 h was allowed, to increase the number of evaluable samples. Additionally, these samples from aCAD patients were kept at room temperature, since this reduces cell death and subsequent lack in responsiveness upon stimulation (data on file).

### Safety Assessments

Safety assessments after in vivo exposure were performed at regular intervals during the follow up period. These consisted of vital signs, 12-lead electrocardiograms (ECGs), physical examination, registration of adverse events (AEs), and routine clinical chemistry and haematology evaluation. Safety blood samples were collected and analysed in accordance with local protocols.

### Cytokines

TNF-α and IL-6 were quantitated in culture supernatants and in plasma samples by Good Biomarker Sciences (GBS, Leiden, The Netherlands). For supernatants a R&D Quantikine ELISA assay (R&D Systems, Inc., Minneapolis, United States) was used, and for plasma samples a R&D Quantikine HS ELISA assay. All samples from one subject were assayed in one run. LPS stimulated samples were initially measured after 20-fold and 50-fold dilution for TNFα and IL-6 respectively in the manufacturer provided diluents. Other samples were initially measured undiluted. Samples were remeasured with higher dilution as needed.

TNF-α and IL-6 values in the supernatants were accepted when duplicates were <20% CV for values within the calibration range: LLOQ (lower limit of quantitation) 15.6 pg/mL and 3.1 pg/mL, and ULOQ (upper limit of quantitation) 1000 pg/mL and 300 pg/mL, for TNF-α and IL-6 respectively. TNF-α and IL-6 values in the plasma samples were accepted when duplicates were <20% CV for values within the calibration range: LLOQ 0.5 pg/mL and 0.16 pg/mL, and ULOQ 32 pg/mL and 10 pg/mL, for TNFα and IL-6 respectively.

### Statistical Analysis

All available data were included in the analyses unless otherwise indicated. Values <LLOQ or >ULOQ were replaced by respectively the LLOQ or ULOQ, as appropriate. Log-normally distributed parameters were ln-transformed prior to analysis.

Individual ratios of stimulated cytokine levels over unstimulated (background) levels were calculated and compared statistically. In case of background levels <LLOQ or stimulated levels >ULOQ, the corresponding ratio (respectively [stimulated]/LLOQ or ULOQ/[unstimulated]) was regarded as the lower margin of the interval (calculated ratio,∞] for the purpose of categorical data analysis.

Continuous data were primarily analysed using an analysis of variance, which could include a covariance analysis to correct for confounding factors. Contrasts and effects (with 95% confidence intervals) were calculated as relevant according to the Tukey method. If the assumption of equal variance was not met, multiple Welch’s t-tests were performed to evaluate the validity of the statistical results.

Categorical data were analysed in a logistic regression model, which could include a covariance analysis to correct for confounding factors. For contingency tables, marginal homogeneity was tested with the Stuart-Maxwell test.

Data analysis was performed with R (v2.15.2, R Foundation for Statistical Computing, Vienna, Austria, 2012 [R Development Core Team, 2012]). Results are presented as mean (standard deviation or 95% confidence interval) for continuous data and as number (percentage) for categorical data, unless otherwise specified.

## Results

### Ex Vivo Exposure

In total, 35 HV and 35 sCAD patients, who were screened for participation in the MDCO-216 phase I clinical trial, had evaluable results following ex vivo exposure and were included in the analysis. Additionally, 38 aCAD patients signed informed consent and provided a blood sample for ex vivo testing in the prespecified period (October 2013 to May 2014), of whom 35 had evaluable results and were included in the analysis. Baseline characteristics of the different populations are presented in Table 2.

**Table 2:** Population characteristics

	exposed in vivo		exposed ex vivo		
Parameter	HV	sCAD	HV	sCAD	aCAD
	(*n* = 24)	(*n* = 24)	(*n* = 35)	(*n* = 35)	(*n* = 35)
Age (year)	26.2 (8.6)	62.8 (7.0)	24.6 (7.5)	64.0 (7.8)	64.4 (12.9)
Height (cm)	175 (8.7)	177 (6.7)	176 (8.7)	177 (6.3)	176 (10.7)
Body weight (kg)	70.0 (11.3)	85.0 (12.8)	69.8 (10.8)	86.5 (13.3)	81.4 (19.2)
BMI (kg/m2)	22.5 (1.8)	27.0 (3.3)	22.3 (1.9)	27.4 (3.3)	26.1 (5.3)
Gender					
Female (*n*)	14 (58%)	1 (4%)	18 (51%)	1 (3%)	11 (31%)
Male (*n*)	10 (42%)	23 (96%)	17 (49%)	34 (97%)	24 (69%)
Revascularisation	procedure				
CABG (*n*)		12 (50%)		14 (40%)	
PCI (*n*)		12 (50%)		21 (60%)	35 (100%)
Coronary involved					
Cx (*n*)		6 (25%)		10 (29%)	15 (43%)
LAD (*n*)		7 (29%)		8 (23%)	19 (54%)
RCA (*n*)		6 (25%)		12 (34%)	15 (43%)
Unknown (*n*)		10 (42%)		13 (37%)	12 (34%)

Characteristics as mean (standard deviation) or number (percentage) of different populations, who were exposed in vivo to MDCO-216 or who were exposed ex vivo to both MDCO-216 and ETC-216. *aCAD* Patients with acute coronary artery disease (CAD); *BMI* Body mass index; *CABG* Coronary artery bypass grafting; *Cx* Circumflex artery; *HV* Healthy volunteers; *LAD* Left anterior descending artery; *PCI* Percutaneous coronary intervention; *RCA* Right coronary artery; *sCAD* Patients with stable coronary artery disease (CAD).

Table 3 presents the released IL-6 upon stimulation of whole blood samples with either ETC-216 or MDCO-216 in relation to background IL-6 levels. From this table, it is seen that ETC-216 clearly elicits a cytokine response, especially when compared to MDCO-216, which seemingly inhibits spontaneous (unstimulated) cytokine release, with a geometric mean ratio of 0.7–1.0, for respectively sCAD and aCAD. This was not caused by an interference of MDCO-216 in the measurement of IL-6, as was determined by measuring a sample with known cytokine levels with and without spiking of MDCO-216 just prior to analysis.

**Table 3:** Ex vivo results

	HV	sCAD	aCAD
	(*n* = 35)	(*n* = 35)	(*n* = 35)
***IL-6***			
LPS	797.7 (1.9)	933.5 (2.0)	62.1 (6.5)
ETC-216*	15.8 (2.9)	9.5 (3.6)	3.8 (4.0)
MDCO-216	0.8 (1.9)	0.7 (1.5)	1.0 (2.0)
***TNF-α***			
LPS	117.4 (1.9)	242.0 (1.8)	46.9 (4.0)
ETC-216*	9.0 (2.8)	6.0 (3.8)	3.5 (4.0)
MDCO-216	0.8 (1.8)	0.7 (1.9)	1.0 (2.0)

Geometric mean (standard deviation) of ratios of LPS, ETC-216 and MDCO-216 stimulated cytokine concentration over unstimulated (background) levels for IL-6 and TNF-α

*Ratios are underestimated as a result of stimulated samples being >ULOQ: for IL-6 in 26 (74.3%) HV, 19 (54.3%) sCAD, 16 (45.7%) aCAD; and for TNF-α in 20 (57.1%) HV, 24 (68.6%) sCAD, 6 (17.1%)

*aCAD* Patients with acute coronary artery disease (CAD); *HV* Healthy volunteers; *LPS* Lipopolysaccharide; *sCAD* Patients with stable coronary artery disease (CAD); *ULOQ* Upper limit of quantitation

Because many ETC-216 stimulated samples had cytokine concentrations >ULOQ (e.g. HV: 26 [74.3%] for IL-6 and 20 [57.1%] for TNF-α), preventing the use of an analysis of variance, the ratios of ETC-216 and MDCO-216 stimulated over background levels were categorised and marginal homogeneity of the contingency table of MDCO-216 versus ETC-216 was statistically tested with the Stuart-Maxwell test. Categories were chosen as <0.2, [0.2,0.5), [0.5,1), [1,2), [2,5), ≥5, based on the fact that virtually all >ULOQ values resulted in a ratio greater than 5. For IL-6 the thus obtained *p*-values under the null hypothesis of marginal homogeneity were <10^−5^, <10^−5^, and 0.0004 for HV, sCAD, and aCAD, respectively; the corresponding results for TNF-α were <10^−5^, <10^−4^, and 0.0005, indicating that ETC-216 and MDCO-216 yielded statistically significantly different cytokine responses.

When comparing the different populations, ETC-216 generally induced lower IL-6 release in the CAD patients than in healthy volunteers. Lower ratios in the CAD populations were also obtained for the MDCO-216 stimulated samples, but LPS exposure resulted in a lower ratio in the aCAD population only. These differences could not be related to age, weight, BMI, blood pressure, (differential) leucocyte count, the severity of the CAD based on total obstruction, the coronary involved, or the type of revascularisation procedure.

The ex vivo results in the aCAD population did not substantially differ from those obtained in the sCAD population. However, some acute CAD patients had considerably higher background (unstimulated) IL-6 levels, up to 2664 pg/mL (median 37.3 pg/mL), compared to a maximum of 84.4 pg/mL (median 14.1 pg/mL) in sCAD. A relationship could not be detected between background levels and demographics or clinical parameters, such as severity of the coronary syndrome based on total coronary obstruction as estimated during acute angiography.

Bioanalytical causes for this phenomenon were not found, neither could the high background levels be related to the interval between sample collection and processing. Albeit true that a time dependent decrease in cytokine response was observed, a prolonged interval was not associated with higher background levels, and judging from the ratios, the overall effects of ETC-216, MDCO-216, and LPS in these samples were consistent with the results obtained from samples that were processed immediately after collection (data not shown).

Statistical significance was reached for the population differences in MDCO-216 and LPS stimulated results, based on an analysis of variance of the ln-transformed ratios over background (unstimulated) cytokine levels, which included sex as covariate. For LPS, the difference in TNF-α ratios between the sCAD and HV population was 206% (95% confidence interval [CI]: 120–355, *p* = 0.0057), between aCAD and HV 40% (24–68, *p* = 0.0002), and between aCAD and sCAD 19% (11–33, *p* < 10^−9^). For MDCO-216 only the difference between sCAD and aCAD populations was statistically significant (IL-6: ratio 69% [95%-CI: 50–95], *p* = 0.0170; TNF-α: ratio 67% [48–94], *p* = 0.0169). Other covariates were not found to have a statistically significant effect, nor improved the model fit.

Population differences were difficult to test statistically for the ETC-216 stimulated condition and IL-6 results following LPS stimulation, due to many stimulated cytokine levels being >ULOQ. Analysing a reduced dataset which only included values <ULOQ resulted in underpowered comparisons. Attempts to fit a multinomial regression model after categorising the data failed for the same reason.

### In Vivo Exposure

Table 2 lists the baseline characteristics of the populations exposed to MDCO-216 in vivo. The most commonly used medication by stable CAD patients was an antithrombotic agent (96%), mainly acetylsalicylic acid (83%). Statins were used by 92%, β-blockers by 58%, and ACE-inhibitors by 38% of the patients.

In the HV population exposed to MDCO-216, two subjects complained of abdominal pain or distension, one (50%) in the 10 mg/kg group and one (25%) in the 20 mg/kg group, which started 9 h after administration. Stable CAD patients receiving MDCO-216, reported nausea once (4 h post dose, 20 mg/kg group) and diarrhoea once (13 h post dose, 40 mg/kg). The latter case was a patient who had undergone a cholecystectomy and since then regularly developed diarrhoea after ingesting high-fat meals.

No inflammatory reaction was observed in HV and sCAD patients, judging by body temperature, white cell counts, neutrophil counts, C-reactive protein, and circulating cytokines (Fig. 1).

## Discussion

Recombinant proteins represent a powerful class of drugs that is employed to supplement absent or insufficient quantities of essential enzymes, hormones, and coagulation factors. Additionally, peptides can be designed to specifically interact with cells or receptors and thus interfere in the pathophysiology of certain diseases [11–13].

However, since these proteins or peptides are invariably expressed in allogeneic, often non-human, cell systems foreign material is released into the medium together with the protein of interest. Countless impurities can trigger the immune system; especially bacterial based platforms, such as those using *Escherichia coli*, are notorious suppliers of immunostimulatory impurities like endotoxin (lipopolysaccharide or LPS) [14]. Also the remaining proteins in a pharmaceutical, collectively referred to as host cell proteins (HCP), can potentially elicit an inflammatory reaction [15].

ETC-216, expressed in *E. coli*, was approved for intravenous administration to humans in accordance with all pertaining regulatory guidelines. Specifically, the limulus amebocyte lysate (LAL) test was negative, HCP levels were ≤10 ppm, and each dose contained <10 ng of residual DNA.

Nevertheless, administration of ETC-216 to healthy volunteers induced dose-dependent neutrophilic leucocytosis, increases in body temperature, and gastrointestinal side effects. In CAD patients, hypersensitivity-like reactions were observed as well as gastrointestinal side effects. In retrospect, these findings are easily recognised as signs and symptoms of an inflammatory response caused by the HCP impurities.

After several modifications were made to the manufacturing process to reduce the HCP levels, the recombinant ApoA-I_M_ was reintroduced as MDCO-216. Because it was deemed unethical to expose a human population to a product with a known immunostimulatory propensity (ETC-216), an ex vivo whole blood incubation assay was applied to compare differences in cytokine response to MDCO-216 and ETC-216.

Results demonstrated that ETC-216 clearly induced cytokine release, in contrast to MDCO-216. In HV and sCAD patients, MDCO-216 even slightly inhibited (spontaneous) release. This difference was statistically significant for all populations, with *p*-values ≤0.0005. The observed inhibition of approximately 30% by MDCO-216 is in accordance with previous studies that have reported that HDL or HDL-like particles display many anti-inflammatory properties [16].

The absence of an inflammatory reaction was confirmed in a clinical trial with MDCO-216. No increases in neutrophils were observed following MDCO-216 infusion in HV and sCAD patients. Also, the more sensitive biomarkers (CRP and circulating cytokines) did not suggest immune stimulation by MDCO-216. Additionally, the observed gastrointestinal side effects did not display a dose-relationship as was seen for ETC-216, although it should be noted that MDCO-216 was infused at a lower rate compared to ETC-216.

An interesting question to be asked is whether an ex vivo incubation assay can be implemented to detect adverse immune stimulation. Certainly, a whole blood stimulation test can detect a myriad of pyrogenic substances, such as LPS, porins, lipoteichoic acid (LTA) and peptidoglycan [17–22]. Nonetheless, many uncertainties still surround the interpretation of its result [23]. Our results revealed differences in reactivity between the three tested populations, not only with regard to the HCP impurities in ETC-216, but also to LPS, which highlights some of the uncertainties.

For example, LPS stimulation resulted in higher cytokine responses in sCAD patients compared to HV, and ETC-216 induced lower levels in sCAD patients compared to HV. After correction for the differences in monocyte count, the statistically significant differences remained. Other factors, such as age, BMI, and blood pressure could not explain any of the found effects.

Comorbidities may have influenced the response, as – for example – hypertension was previously found by Dörffel et al. [24] to increase TNF-α and IL-1β secretion from peripheral blood monocytes after in vitro LPS stimulation by >50%, although these patients were untreated. The antihypertensives losartan, captopril, and amlodipine dose-dependently reduced IL-1β release induced by LPS, but not below LPS stimulated levels in normotensive subjects [25].

Other cardiovascular medications are also known to influence the (innate) immune response. For example, certain calcium channel blockers were observed to interfere with both flagellin and LPS signalling [26, 27]. Statins and aspirin demonstrate similar anti-inflammatory properties [28–30]. Interestingly however, whereas many cardiovascular drugs inhibit TLR-4 mediated responses, with β-blockers being a notable exception [31, 32], ex vivo LPS stimulation induced higher cytokine levels in sCAD patients than in HV. Although effects of certain cardiovascular medications and conditions on toll-like receptor (TLR) signalling have not been examined as extensively for TLR-5 (flagellin) as for TLR-4 (LPS), our results suggest that the response to flagellin and other HCP impurities can be modified by these factors as well.

Notwithstanding the aforementioned effects, compared to stable CAD patients, acute CAD patients displayed higher background cytokine levels, as well as an overall diminished responsiveness to both LPS and ETC-216. Severity of the coronary disease, based on total obstruction, or the coronary involved was not related to background cytokine levels, or any of the observed effects after ex vivo stimulation with LPS, ETC-216 or MDCO-216.

ACS is associated with elevated plasma levels of pro-inflammatory cytokines, chemokines, and leucocytes, which are governed at least partly by TLR-4 stimulation [33, 34]. Conversely, stress hormones such as catecholamines and hydrocortisone, that have a general immune-inhibiting mode of action, are increased in parallel [35–37]. This combination might explain both the high background cytokine levels and the reduced response to ETC-216 observed in the aCAD patients. TLR-4 mediated cytokine release in ACS can also account for the observation that a subsequent (experimental) LPS challenge did only modestly increase IL-6 and TNF-α concentrations ex vivo.

It should be stressed, however, that although (the consequences of) an inflammatory response can be influenced by external factors, a reduced response is not synonymous with an improved outcome, especially in critically ill patients. This warrants a cautious approach when exposing (vulnerable) humans to an experimental biologic. Furthermore, it underlines the current limitations of an ex vivo stimulation test in reliably predicting inflammatory reactions upon in vivo administration in the target population, although it can be used to highlight differences between two pharmacological products within a population.

Concluding, MDCO-216 does not elicit an acute immune response in healthy volunteers nor in patients with stable coronary artery disease, in contrast to what was previously observed with ETC-216. Results from an ex vivo stimulation with both products suggests the same holds true for patients with an acute coronary syndrome.
